# A preliminary quantitative proteomic analysis of glioblastoma pseudoprogression

**DOI:** 10.1186/s12953-015-0066-5

**Published:** 2015-03-12

**Authors:** Peng Zhang, Zhengguang Guo, Yang Zhang, Zhixian Gao, Nan Ji, Danqi Wang, Lili Zou, Wei Sun, Liwei Zhang

**Affiliations:** Department of Neurosurgery, Beijing Tiantan Hospital, Capital Medical University, No. 6 TiantanXili, Dongcheng District, Beijing, 100050 China; Core Facility of Instrument, Institute of Basic Medical Sciences, Chinese Academy of Medical Science/School of Basic Medicine, Peking Union Medical College, No. 5 Dongdan Santiao, Dongcheng District, Beijing, 100005 China

**Keywords:** iTRAQ labeling, Pseudoprogression, Quantitative proteomics

## Abstract

**Backgrounds:**

Pseudoprogression disease (PsPD) is commonly observed during glioblastoma (GBM) follow-up after adjuvant therapy. Because it is difficult to differentiate PsPD from true early progression of GBM, we have used a quantitative proteomics strategy to identify molecular signatures and develop predictive markers of PsPD.

**Results:**

An initial screening of three PsPD and three GBM patients was performed, and from which 530 proteins with significant fold changes were identified. By conducting biological functional analysis of these proteins, we found evidence that the protein synthesis network and the cellular growth and proliferation network were most significantly affected. Moreover, six of the proteins (HNRNPK, ELAVL1, CDH2, FBLN1, CALU and FGB) involved in the two networks were validated (n = 18) in the same six samples and in twelve additional samples using immunohistochemistry methods and the western blot analysis. The receiver operating characteristic (ROC) curve analysis in distinguishing PsPD patients from GBM patients yielded an area under curve (AUC) value of 0.90 (95% confidence interval (CI), 0.662-0.9880) for CDH2 and.0.92 (95% CI, 0.696-0.995) for CDH2 combined with ELAVL1.

**Conclusions:**

The results of the present study both revealed the biological signatures of PsPD from a proteomics perspective and indicated that CDH2 alone or combined with ELAVL1 could be potential biomarkers with high accuracy in the diagnosis of PsPD.

**Electronic supplementary material:**

The online version of this article (doi:10.1186/s12953-015-0066-5) contains supplementary material, which is available to authorized users.

## Introduction

Glioblastoma (GBM) is one of the most malignant brain tumors. After the postoperative use of radiotherapy for GBM became common, a phenomenon termed pseudoprogression disease (PsPD) was identified [1,2]. With the widely implementation of the Stupp protocol for treating GBM, this phenomenon has been inceasingly reported, with an incidence rate varies among reports (5.5%-64%) [3-6]. PsPD is often misdiagnosed as tumor recurrence and misleads the clinical treatment. However, little is known about why PsPD occurs in a subset of GBM patients and the fundamental biological features of PsPD remain unclear [5,7-10].

From a diagnostic perspective, no single imaging technique, including T1-weighted magnetic resonance imaging (MRI), magnetic resonance spectroscopy (MRS), relative cerebral blood volume (rCBV)-based parametric response mapping and ^18^fluorodeoxyglucose (^18^ F-FDG)-positron emission computed tomography (PET), has been adequate for differentiating PsPD from true early tumor progression with high sensitivity and specificity [4,5,11-16]. Moreover, molecular biological studies have failed to uncover biomarkers linked to PsPD for clinical use. Although a multitude of genetic and molecular changes involved in GBM, including O6-methylguanine–DNA methyltransferase (MGMT) promoter methylation, isocitrate dehydrogenase 1 (IDH1) mutation, p53 mutation and Ki-67 expression, have been found to be associated with PsPD, the predictive value of these biomarkers remains debatable [5,8,17-19]. Therefore, except for cases of pathological verification, PsPD is still predominantly diagnosed retrospectively. Thus, there is an urgent need for the exploration of more reliable biochemical markers that can accurately identify PsPD.

**Figure 1:**
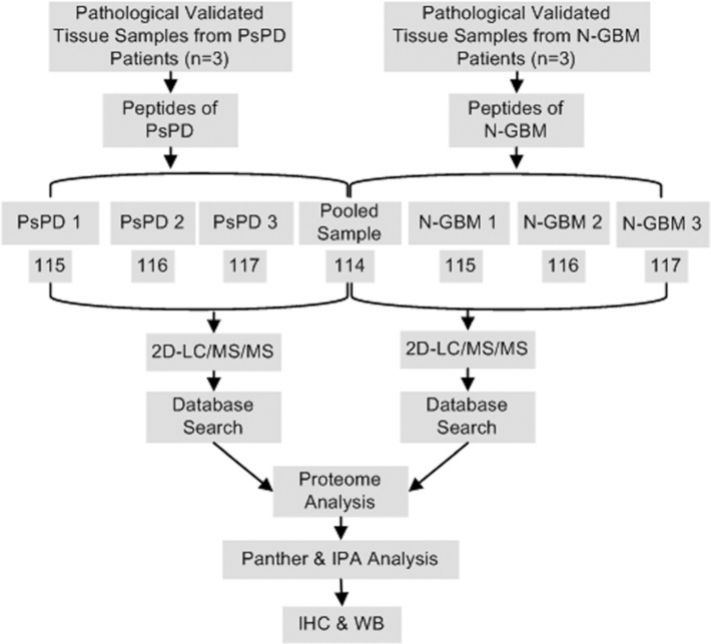
**Workflow of the iTRAQ proteomic strategy.** In this work, three pathologically verified tissue samples of PsPD and three samples of GBM were used for iTRAQ labeled proteomic analysis. The proteins identified were quantitatively analyzed using Panther and IPA for biological functions analysis. Several candidate proteins with interesting biological functions were selected and further validated using IHC and WB of the same samples used for proteomic analysis as well as additional samples.

Proteomic measurements provide a wealth of biological information and several proteomic studies of gliomas have been recently reported [20,21], which demonstrated a possibility to investigate this phenomenon by using proteomics methods. Herein, this present study was designed to identify biological signatures and explore biomarkers for PsPD using differential proteomic techniques (Figure 1).

## Results

### Identification of proteins with significant fold changes in PsPD versus GBM

**Figure 2:**
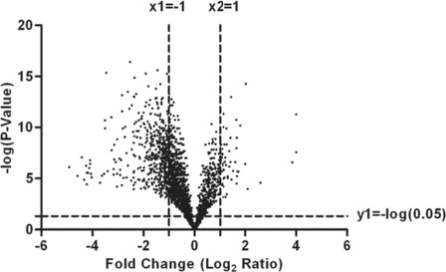
**Volcano plots of identified proteins in PsPD vs GBM.** The x-axis of the graph refers to the log transformation of fold change, whereas the y-axis of the graph refers to the negative log transformation of the p-value.

In this iTRAQ-labeling proteomic study, by comparing the total proteomes of tissue from PsPDs with the proteomes of tissues from GBMs, we identified 4048 proteins in PsPD and 3846 proteins in GBM (Additional file 1: File s1, Additional file 2: File s2, Additional file 3: File s3 and Additional file 4: File s4). To measure the quantitative correlation between pairwise sample combinations within each group, a Pearson’s correlation coefficient (ranged from 0.967 to 0.980) was calculated and showed high biological reproducibility (Additional file 5: Figure s1). To maintain a low false-positive rate of comparative analysis between the groups, an average CV of 0.37 (Additional file 5: Figure s2) was employed to filter out data with poor linearity, corresponding to coverage of more than 80% of the 3390 quantified proteins both in PsPDs and GBMs. Next, a threshold of ≥2-fold and p < 0.05 was taken to identify 530 proteins with significant fold changes for further analysis (Figure 2). Among these proteins, 57 proteins were up-regulated and 473 were down-regulated in PsPD compared with GBMs (Additional file 6: File s5 and Additional file 7: File s6).

### Interaction networks and functional pathway analysis

**Figure 3:**
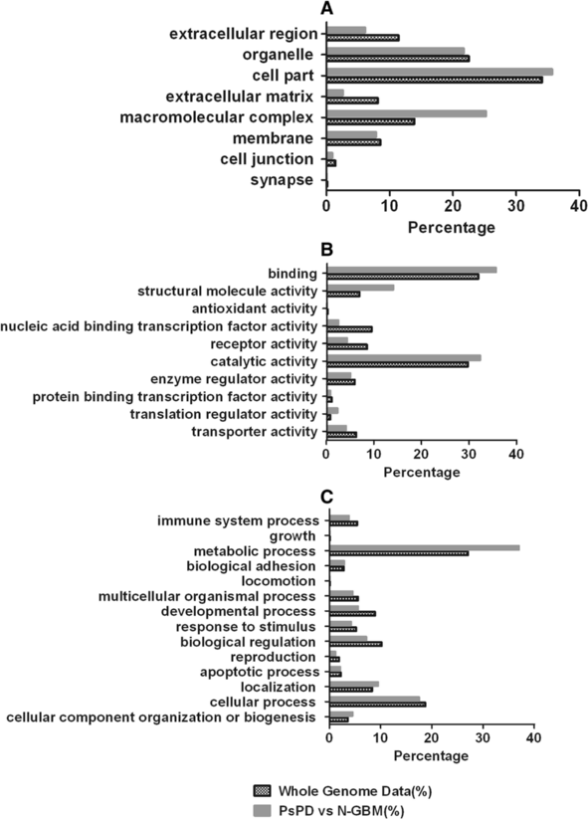
**Panther analysis of PsPD vs N-GBM.** Graph **A** shows cellular compartment analysis; Graph **B** shows molecular function analysis; and Graph **C** shows biological process analysis.

**Figure 4:**
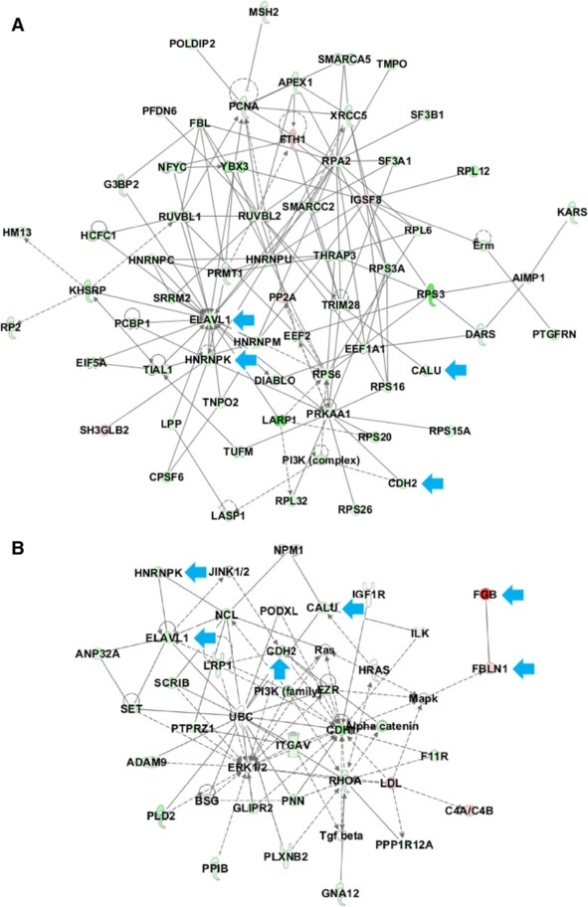
**Cellular growth and proliferation network and protein synthesis network from IPA analysis.** Graph **A** shows the protein synthesis focused network, and Graph **B** illustratescellular growth and proliferation focused function network. Proteins in red were up-regulated in PsPD compared with N-GBM, and proteins in green were down-regulated in PsPD compared with N-GBM. Proteins pointed by the blue arrow were the selected out candidate proteins used for analysis and further validation.

Functional pathway analysis was performed for the 530 proteins to better understand the biological features of PsPD. Gene ontology analysis indicated broad distribution of these proteins, with the most frequently represented categories being cellular compartment, molecular function, and biological processes (Figure 3). The results of Ingenuity Pathway Analysis (IPA) analysis indicated that the protein synthesis network and the cellular growth and proliferation network were mostly affected (Figure 4),with a series of cellular functions being significantly inhibited in PsPD compared with GBM (Additional file 5: Figure s3). For example, the invasion (z-score:-2.575) and proliferation (z-score:-2.886) abilities of tumor cells were significantly downregulated in PsPD compared with GBM. Moreover, the translation (z-score: −2.464), synthesis of protein (z-score: −2.236) and metabolism of protein (z-score:-2.046) were also significantly downregulated in PsPD compared with GBM.

### Selection of candidate proteins for validation

**Figure 5:**
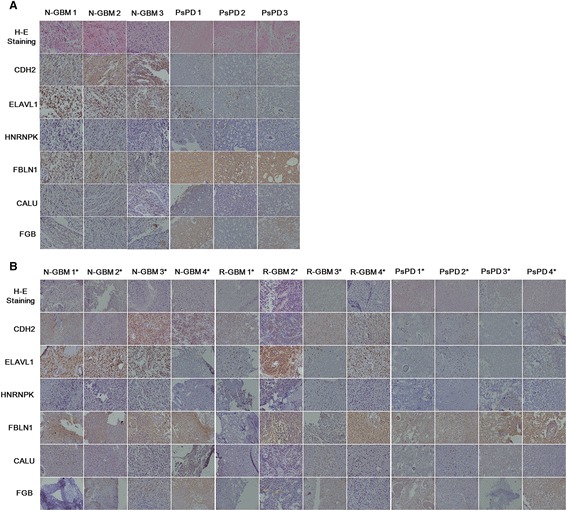
**Results of immunohistochemical analysis of CDH2, ELAVL1, HNRNPK, FBLN1, CALU and FGB in tissue samples.** Magnification: 200X. Representative images of paraffin-embedded sections of PsPD and GBM tissue that were HE stained and immunostained for CDH2,ELAVL1, HNRNPK, FBLN1, CALU and FGB. Graph **A** shows the validation of these six candidate proteins in the six samples used for proteomic analysis. The first three columns show the validation results in N-GBMs and the second three columns show the results in the PsPDs. Graph **B** shows the validation in an additional twelve samples. The first four column shows the validation results in additionally selected N-GBMs, the second four column shows the results in R-GBMs, and the third four column shows the results in PsPDs. * indicates the twelve additionally selected samples.

**Figure 6:**
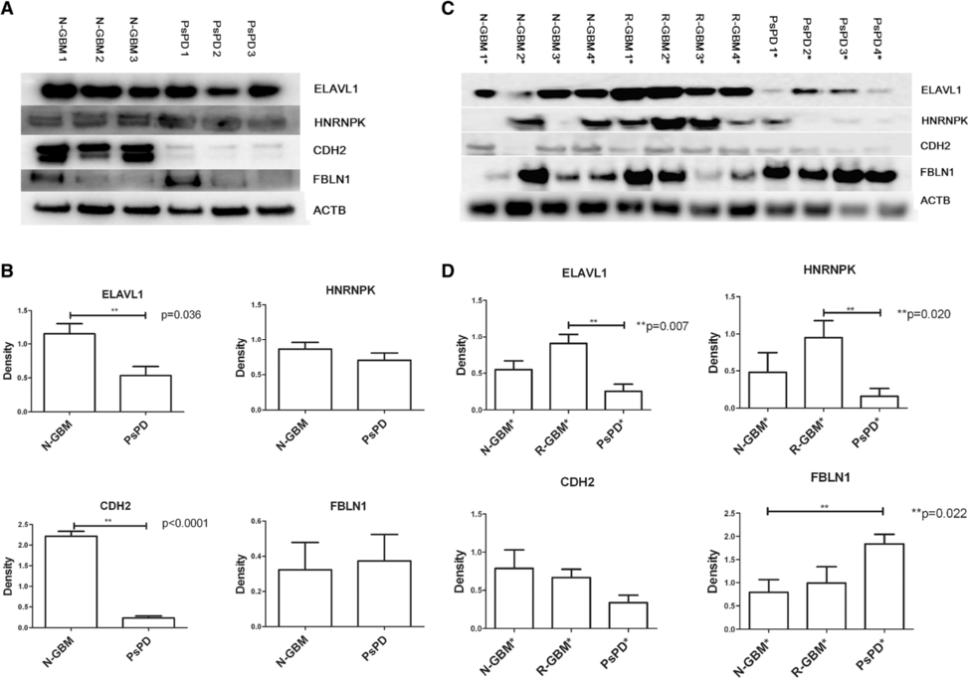
**Western blot analysis for ELAVL1, HNRNPK, CDH2 and FBLN1 in tissue samples.** Graph **A** shows that high levels of ELAVL1, HNRNPK, CDH2 and low levels FBLN1 were detected in N-GBMs compared with PsPDs in the six samples for proteomic analysis. Graph **B** shows the quantification of expression levels using densitometry. Graph **C** shows that high levels of ELAVL1, HNRNPK, CDH2 and low levels of FBLN1 were detected in GBMs (both N-GBM and R-GBM) compared with PsPDs in additional twelve samples. Graph **D** shows the quantification of expression levels using densitometry. * indicates the twelve additionally selected samples;** p < 0.05.

Three candidate proteins (HNRNPK, ELAVL1 and CDH2) involved in the two networks and acting as key-point proteins were selected out. In order to explore more promising biomarkers, all secreted proteins with more than 2 fold changes (Additional file 8: File s7) were searched against the protein atlas database (http://www.proteinatlas.org), because the protein atlas database provided the expression levels of candidate proteins in specific tissues and related antibodies. Proteins with median or high positive expression in glial cell or tissue were chosen for further functional analysis. Three proteins (FBLN1, CALU and FGB) meeting the criteria were selected out. The results of IHC and WB validation of the six proteins were in accordance with the proteomic findings (Figures 5, 6). Moreover, a quantitative analysis of the WB results was performed (Table 1, Figure 6). As shown in the figure, statistically significant differences were found between the groups.

**Table 1:** **Candidate proteins used for validation and details**

**Candidate Proteins**	**Accession Number**	**iTRAQ**		**IHC (PsPD vs N-GBM)**	**WB Quantitative Analysis**		
		**FC (PsPD vs N-GBM)**	**P value**		**PsPD vs N-GBM (MS)**	**PsPD vs N-GBM** ^**#**^	**PsPD vs R-GBM** ^**#**^
HNRNPK	P61978	0.34	**	down-regulation	0.82	0.26	0.14*
ELAVL1	B4DVB8	0.23	**	down-regulation	0.46*	0.33	0.21*
FBLN1	P23142	2.74	**	up-regulation	1.19	1.63*	1.43
CDH2	P19022	0.29	0.05	down-regulation	0.1*	0.35	0.4
CALU	O43852	0.33	0.15	down-regulation	No Validation	No Validation	No Validation
FGB	P02675	16.06	**	up-regulation	No Validation	No Validation	No Validation

Note: The 1st column refers to the candidate proteins used for validation; the 2nd column refers to the corresponding accession number of these candidate proteins; the 3rd column refers to the results of iTRAQ labeled quantitative analysis, FC, fold change, **p < 0.01; The 4th column refers to the differential expression levels of these candidate proteins in the immunohistochemistry (IHC) analysis; The 5th column refers to the results of the western blot validation of the 4 candidate proteins, ELAVL1, HNRNPK, CDH2 and FBLN1, ^#^indicates the results in additional samples, *p < 0.05.

### Evaluation of HNRNPK, ELAVL1, CDH2 and FBLN1 as diagnostic markers for PsPD

**Figure 7:**
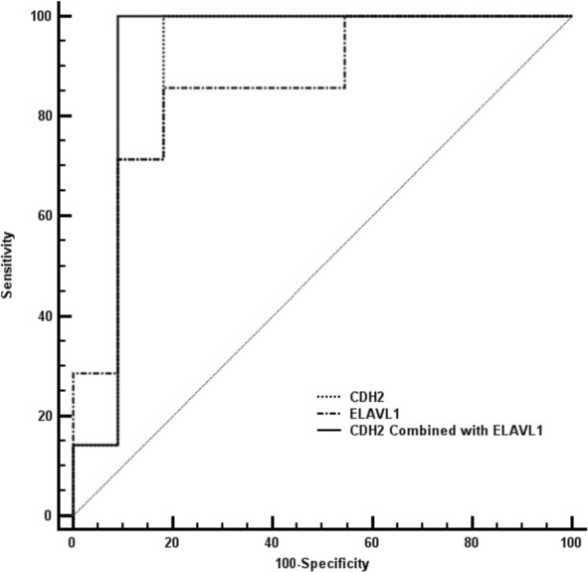
**Roc curve of predictive biomarkers.** The ROC curve of CDH2, ELAVL1 and the combination of these two candidate proteins was shown in the graph with different lines.

The WB analysis revealed that HNRNPK, ELAVL1, CDH2 and FBLN1 were of statistical significance and exihibited obvious fold changes between PsPDs and GBMs (Table 1). Furthermore, the area under the ROC curves for ELAVL1, HNRNPK, CDH2 and FBLN1 were 0.86 (p = 0.013), 0.75 (p = 0.077), 0.90 (p = 0.006) and 0.66 (p = 0.258), respectively (Figure 7, Additional file 9: Table S1). A pairwise comparison of ROC curves shows no statistical difference between these four proteins (Additional file 9: Table S2). Furthermore, the area under the combined ROC curve for CDH2 and ELAVL1 was 0.92 (P = 0.003), indicating that the diagnostic value of CDH2 alone or combined with ELAVL1 was improved.

## Discussion

By using iTRAQ-labeled proteomic analysis and conducting further biological functional analysis of fold-changed proteins, we identified the biological features of PsPD from the perspective of proteomics and explored several candidate proteins to be predictive biomarkers.

### Protein metabolism and upstream regulatory mechanisms play fundamental roles

The results of the biological analysis revealed the protein synthesis network to be broadly affected. Based on the data from the present study, the expression level of proteins involved in protein synthesis and upstream regulatory mechanisms, such as RNA post-transcriptional modification, post-translational modification and protein folding are significantly different between PsPDs and GBMs (Figure 4, Additional file 5: Figure s3, Additional file 7: File s5 and Additional file 8: File s6), indicating these mechanisms may be significantly affected. Two candidate proteins, HNRNPK and ELAVL1, involved in the protein synthesis network were selected and validated.

HnRNPs comprise a large family of proteins with approximately 30 members that share some structural domains. Previous studies have shown that hnRNPs played central roles in several cellular functions, among which HNRNPK was found to play an essential role in cellular proliferation by regulating protein synthesis and is over-expressed in head and neck tumors [22,23]. In recent studies, HNRNPK was also found to play a significant role in the mechanism of DNA damage-related cell cycle arrest under ionizing conditions [24,25], which is similar to the effect of radiotherapy. In the present study, hnRNPs (HNRNPC, HNRNPK, HNRNPM and HNRNP) were found to play roles in the protein synthesis network and were down-regulated in PsPDs compared with GBMs, which may reflect the effect of chemo-radiotherapy treatment in GBM patients.

In addition to the hnRNPs, another RNA-binding protein, ELAVL1, was selected. Under hypoxia, ELAVL1 plays a significant role in the regulation of angiogenesis by stabilizing vascular endothelial growth factor A (VEGF-A) mRNA [26,27]. VEGF-A is one of the major mediators of vascular proliferation in astrocytic tumor [28]. Both VEGF and ELAVL1 were identified down-regulated in PsPD compared with GBM, suggesting the possibility of angiogenesis inhibition in PsPD. This result may also help explain how hypoxia is involved in the formation of PsPD, as has been proposed in several studies [18,29].

### Cellular function interference

Many researchers have proposed that PsPD occurs due to the induction of cell death by radiotherapy and/or chemotherapy of malignant glioma [17,30]. These findings indicate a hypothesis that an underlying relationship between PsPD occurrence and cell death induction by adjuvant therapy may exist [30]. In this present study, the results of biological analysis shows that most of the proteins related to the cellular growth and proliferation functions as well as the invasion and proliferation abilities of tumor cells were down-regulated (Figure 4, Additional file 5: Figure s3, Additional file 6: File s5 and Additional file 7: File s6), demonstrating these functions may have been significantly inhibited. Except for HNRNPK and ELAVL1, another two candidate proteins, CDH2 and CALU, involved in the network of cellular growth and proliferation were selected and validated.

A previous study on brainstem glioma showed that higher expression of CDH2 predicts the progression of malignant tumors and tends to predict a shorter survival time of patients [31]. Other studies also indicated CDH2 may be functionally correlated with tumorigenesis in glioma cells and involved in mediating glioma cell migration [32-34]. In the present study, CDH2 is involved in several cellular functions (Additional file 5: Figure S3, Additional file 9: Table S3) and found to be down-regulated in PsPDs compared with GBMs (Table 1, Figure 4). The results were in accordance with previous studies and may demonstrate the malignancy changes in PsPD.

Another protein CALU, is a calcium-binding protein located in the endothelium that is involved in protein folding and sorting. This protein was recently found to be highly expressed in normal neural stem cells and GBM stem-like cells compared with the GBM tumor tissue [35]. Additionally, the gene CALU was also observed to be up-regulated in GBM but not in low-grade astrocytoma or oligodendroglioma [36]. These results indicated that the expression levels of CALU may be correlated with tumor cell proliferation ability, which is in accordance with the biological analysis results of this present study.

### Validation of secretory proteins as candidate biomarkers

At present, there are no suitable specific biomarkers that can be used to accurately differentiate PsPDs from GBMs. Secretory proteins have the potential to be detected as biomarkers in body fluids. Therefore, we also selected three candidate secretory proteins, CALU (described above), FGB and FBLN1, for validation. The validation results were in accordance with the proteomic findings. It is noteworthy that, previous studies have reported that FBLN1 expression is elevated in breast tumors [37] and ovarian cancer cells [38]. But no details about the roles of FBLN1 in gliomas have been reported previously.

Taken together, the proteomic results as well as the validation results both identified that the expression level of HNRNPK, ELAVL1, CDH2 and FBLN1 in PsPDs were significantly different from GBMs (Figures 5, 6). ROC curves yielded an AUC value of 0.90 (95% CI, 0.662-0.9880) for CDH2 and.0.92 (95% CI, 0.696-0.995) for CDH2 combined with ELAVL1, which indicated that these two proteins could be potential biomarkers with relatively high accuracy in the diagnosis of PsPD.

## Conclusion

In summary, our work offers an initial description of the proteins conserved in PsPDs and GBMs as well as novel information on proteins that are differentially expressed between groups. Through biological analysis and validation of the proteomic findings, this present study not only revealed the molecular signatures but also provide novel markers that may help to identify the mechanisms behind and allow the diagnosis of PsPD. However, due to the low number of samples used in the present study, above conclusions were just preliminary results, therefore, it should be careful to use our conclusions. Further verification in additional samples should be helpful and essential to understand the process.

## Materials and methods

### Sample collection and pathological examination

A set of fresh frozen tissue samples that included PsPD (n = 3) and newly diagnosed GBM (N-GBM, n = 3) was obtained under an Institutional Review Board-approved protocol at the Beijing Tiantan Hospital of Capital Medical University. Consents of clinical data and samples used for the study have been obtained from the patients and their families. PsPD was diagnosed according to the criteria of Macdonald [39] without viable tumor recurrence by pathological verification. The tissue samples were snap-frozen immediately after resection and stored at −80°C. To ensure that the fragments used for proteomic analysis contained a sufficient proportion (at least 80%) of the target tissue, we evaluated each specimen before use. Moreover, twelve additional samples were selected for verification by IHC and WB, including four PsPD, four N-GBM and four recurrent GBM (R-GBM) tissue samples (Additional file 9: Table S4).

### ITRAQ sample preparation

First, 80 mg samples from each of the six frozen tissue samples selected for the proteomics screening were rinsed with PBS, and each sample was then mixed with lysis buffer (50 mMTris-HCl, 2.5 M thiourea, 8 M urea, 4% CHAPS, 65 mM DTT) for total protein extraction. The total protein concentration of each sample was determined using the Bio-Rad RC DC Protein Assay.

The proteins from each sample were pooled equally according to the total amount of protein and digested by filter-aided sample preparation combined with a microwave-assisted protein preparation method as previously described [40,41]. The peptides were dried by vacuum centrifugation and stored at −80°C.

The digested PsPD and GBM samples were mixed equally to create the internal standard and labeled by 114 iTRAQ. The three PsPD samples and the three GBM samples, were individually labeled with 115, 116 or 117 iTRAQ according to the manufacturer’s protocol (ABsciex).

### 2D-LC and MS/MS conditions

For offline separation a HPLC from Waters was used, and for online LC/MS/MS analysis a nano-ACQUITYUPLC system from Waters was used. First, the pooled mixture of the labeled samples was fractionated using a high-pH RPLC column from Waters (4.6 mm × 250 mm, C18, 3 μm). For each fraction the injection volume was 8uL. The samples were loaded onto the column in buffer A1 (1‰ aqueous ammonia in water, pH = 10), and eluted by buffer B1 (1‰ aqueous ammonia in 10% water and 90%ACN; pH = 10, flow rate = 1 mL/min) with the gradient of 5–90%for 60 min. The eluted peptides were collected at a rate of one fraction per minute, and pooled into 20 samples. Each sample was analyzed by LC-MS/MS using an RP C18 self-packing capillary LC column (75 μm × 100 mm, 3 μm) and a Triple TOF 5600 mass spectrometer. For Triple TOF 5600 a nano source was used. The MS data were acquired in high sensitivity mode with detailed parameters for Triple TOF 5600 being set as following: ion spray voltage was 2200v, curtain gas was 25, gas 1 was 5, gas 2 was 0, temperature was 150, declustering potential was 100, mass range was 350–1250 for MS and 100–1800 for MS/MS, collision energy was 35, and the resolution of MS and MS/MS was 40000 and 20000. An elution gradient of 5–30% buffer B2 (0.1% formic acid, 99.9% ACN; flow rate, 0.3 μL/min) for 50 min was used for the analysis. Thirty data-dependent MS/MS scans were acquired for every full scan. The normalized collision energy used was 35%, and charge state screening (including precursors with +2 to +4 charge state) and dynamic exclusion (exclusion duration of 15 s) were performed. Analyst TF 1.6 was used to control the instruments.

### Database search

The MS/MS spectra were searched against the human subset of the Uniprot database (84910 entries) (http://www.uniprot.org/) using the Mascot software version 2.3.02 (Matrix Science, UK). Trypsin was chosen for cleavage with a maximum number of allowed missed cleavages of two. Carbamidomethylation (C) and iTRAQ 4-plex labels were set as fixed modifications. The searches were performed using a peptide and product ion tolerance of 0.05 Da. Scaffold software was used to further filter the database search results using the decoy database method with the following filter: a 1% false-positive rate at the protein level and two unique peptides per protein. After filtering the results as described above, the peptide abundances in different reporter ion channels of the MS/MS scan were normalized. The protein abundance ratio was based on unique peptide results. Proteins with a fold change ≥ 2 were considered significantly altered.

### Bioinformatics analysis

Data filtering was performed according to strict criteria, wherein any missing data values or detection failures were deleted. Pearson’s correlation coefficient was calculated to measure the quantitative correlation among the three biological replicates in each group, and the coefficient of variation within groups was set at CV = 0.37 to filter out low-quality data. A Student’s *t*-test was performed between groups, and differences were considered to be significant when p < 0.05. Any proteins that satisfied the criteria of a fold change (FC) between groups of ≥2 were selected for bioinformatics analysis using Gene Ontology (GO) and Ingenuity Pathway Analysis (IPA).

### GO functional and IPA network analysis

All proteins identified by the two approaches were assigned a gene symbol using the Panther database (http://www.pantherdb.org/). Protein classification was performed based on the functional annotations of the GO project for cellular compartment, molecular functional and biological processed. When more than one assignment was available, all of the functional annotations were considered in the results. Moreover, all of the selected proteins with a significant fold changes were used for pathway analysis using the IPA software (Ingenuity Systems, Mountain View, CA) for network analysis.

### Immunohistochemistry and western blot analysis

IHC was performed on the same six tissue samples used for the proteomic analysis and on twelve additional formalin fixed, paraffin embedded tissue samples. The following primary antibodies were used: anti-ELAVL1mouse monoclonal (Santa Cruz), 1:500; anti-HNRNPK mouse monoclonal (Santa Cruz), 1:50;anti-CDH2rabbit monoclonal (Cell Signaling Technology), 1:250; anti-FBLN1 mouse monoclonal (Santa Cruz),1:125; anti-CALU goat polyclonal (Santa Cruz), 1:100; anti-FGB goat polyclonal (Abcam), 1:16000. After deparaffinization and rehydration, antigen retrieval was performed by immersing the slide in antigenretrieval buffer (10 mM sodium citrate, 0.05% Tween 20, pH = 6.0) at 95°C for 5 min using pressure cooker. Endogenous peroxidases were blocked with 0.03% hydrogen peroxide, and nonspecific binding was blocked with 2% fetal calf serum in Tris-buffered saline with 0.1% Triton X-100 (TBST, pH = 7.6). The sections were then incubated for 1 h at room temperature with primary antibodies followed by peroxidase-labeled polymer conjugate to anti-mouse, anti-rabbit, anti-goat immunoglobulins for 1 h and developed with diaminobenzidine system. The sections were counter stained with the Mayer’s hematoxylin and dehydrated, and the image was taken under microscope.

WBs of the same six samples and additional twelve samples was performed to validate the proteomic quantitation of four selected candidate proteins (HNRNPK, ELAVL1, CDH2 and FBLN1). Proteins extracted from GBM or PsPD tissues were resolved by SDS-PAGE (4–20% gradient precast gel; Invitrogen). The protein bands were electro transferred to a PVDF membrane (Millipore, Bedford, MA), blocked with 2% (v/v) BSA in TBST (150 mM NaCl, 20 mM Tris, 0.1% Tween 20, pH = 7.4) for 2 h at room temperature, followed by incubation with primary antibody (anti-ELAVL1, 1:200 (mouse monoclonal, Santa Cruz); anti-HNRNPK,1:3000 (mouse monoclonal, Santa Cruz); anti-CDH2, 1:800 (rabbit monoclonal, Cell Signaling Technology); anti-FBLN1, 1:100, (mouse monoclonal, Santa Cruz)) diluted with 1% BSA in TBST at room temperature for 2 h. After extensive wash with TBST, the membranes were incubated with horseradish peroxidase-conjugated secondary antibody (anti-mouse or anti-rabbit; EarthOX, USA) diluted with 1% BSA in TBST for 90 min at room temperature. The membranes were developed using Immobilon Western chemiluminescent horseradish peroxidase substrate (Millipore). All the selected proteins ELAVL1, HNRNPK, CDH2 and FBLN1 were validated by Western blot analysis with actin as loading control.

## Additional files

Additional file 1:
**File s1.** Quantitative Peptide List for PsPD Samples. Additional file 2:
**File s2.** Quantitative Peptide List for N-GBM Samples. Additional file 3:
**File s3.** Quantitative Protein List for PsPD Samples. Additional file 4:
**File s4.** Quantitative Protein List for N-GBM Samples. Additional file 5: Figure s1. Pearson correlation coefficient plot of each two proteomic runs related to the tissue specimen in each group. The three graphs in the first row of the figure refers to Pearson coefficient of any two samples in PsPD sample group (ranged from 0.974 to 0.980); The three graphs in the second row of the figure refers to the Pearson coefficient of any two samples in GBM sample group (ranged from 0.967 to 0.978). Additional file 6:
**File s5.** Significantly Fold Changed Proteins between PsPD and N-GBM Samples. Additional file 7:
**File s6.** Quantitative peptides of differentially expressed proteins between PsPD and N-GBM samples. Additional file 8:
**File s7.** List of secreted proteins. Additional file 9: Table S1. Parameters of ROC curve for four proteins.

## Abbreviations

^18^ F-FDG: ^18^fluorodeoxyglucose
CALU: Calumenin
CDH2: N-cadherin
CV: Coefficient of Variance
ELAVL1: Hu-antigen R
FBLN1: Fibulin-1
FC: Fold Change
FGB: Fibrinogen Beta Chain
GBM: Glioblastoma
GO: Gene Ontology
HNRNPK: heterogeneous nuclear ribonucleoprotein K
IDH1: Isocitrate Dehydrogenase 1
IHC: Immunohistochemistry
IPA: Ingenuity Pathway Analysis
MGMT: O6-methylguanine–DNA methyltransferase
MRI: Magnetic Resonance Imaging
MRS: Magnetic Resonance Spectroscopy
N-GBM: Newly Diagnosed Glioblastoma
Panther: Protein Analysis Through Evolutionary Relationships
PsPD: Pseudoprogression Disease
rCBV: Relative Cerebral Blood Volume
R-GBM: Recurrent Glioblastoma
VEGF: Vascular Endothelial Growth Factor
WB: Western Blot
