# Supplementary material for: A preliminary quantitative proteomic analysis of glioblastoma pseudoprogression
Source: Proteome Sci. 2015 Mar 12;13:12. doi: 10.1186/s12953-015-0066-5 (PMC4393599; doi:10.1186/s12953-015-0066-5)

Figure s1 Pearson correlation coefficient plot of each two proteomic runs related to the tissue specimen in each group. The three graphs in the first row of the figure refers to Pearson coefficient of any two samples in PsPD sample group (ranged from 0.974 to 0.980); The three graphs in the second row of the figure refers to the Pearson coefficient of any two samples in GBM sample group (ranged from 0.967 to 0.978).


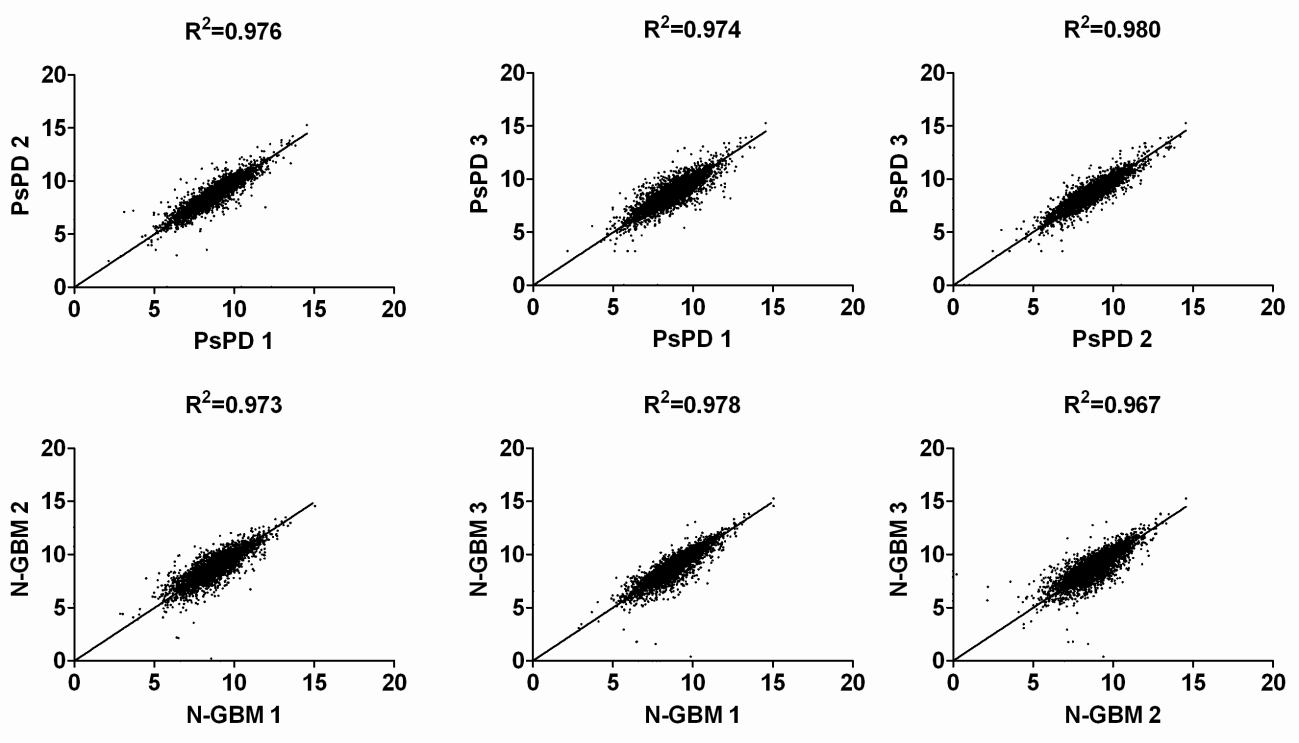


Figure s2 Coefficient of variation of PsPD and GBM. The y-axis refers to the frequency division interval of PsPDs and GBMs respectively. The x-axis refers to the frequency of each division intervals of each sample group.


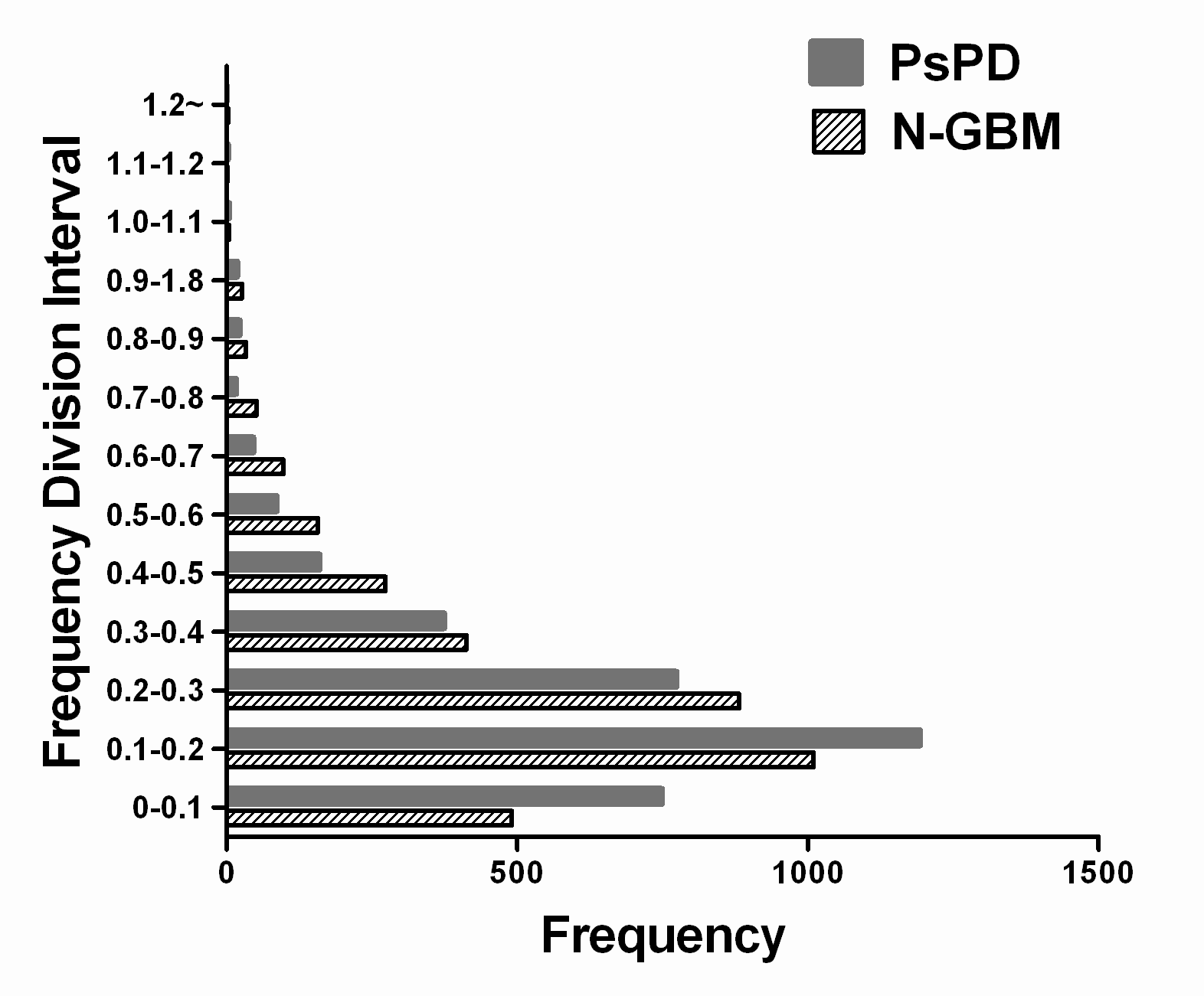


Figure s3 Disease and functional analysis of the 530 significantly fold changed proteins between PsPD and GBM by IPA analysis. The top 20 ranked disease and functional processes were mapped.


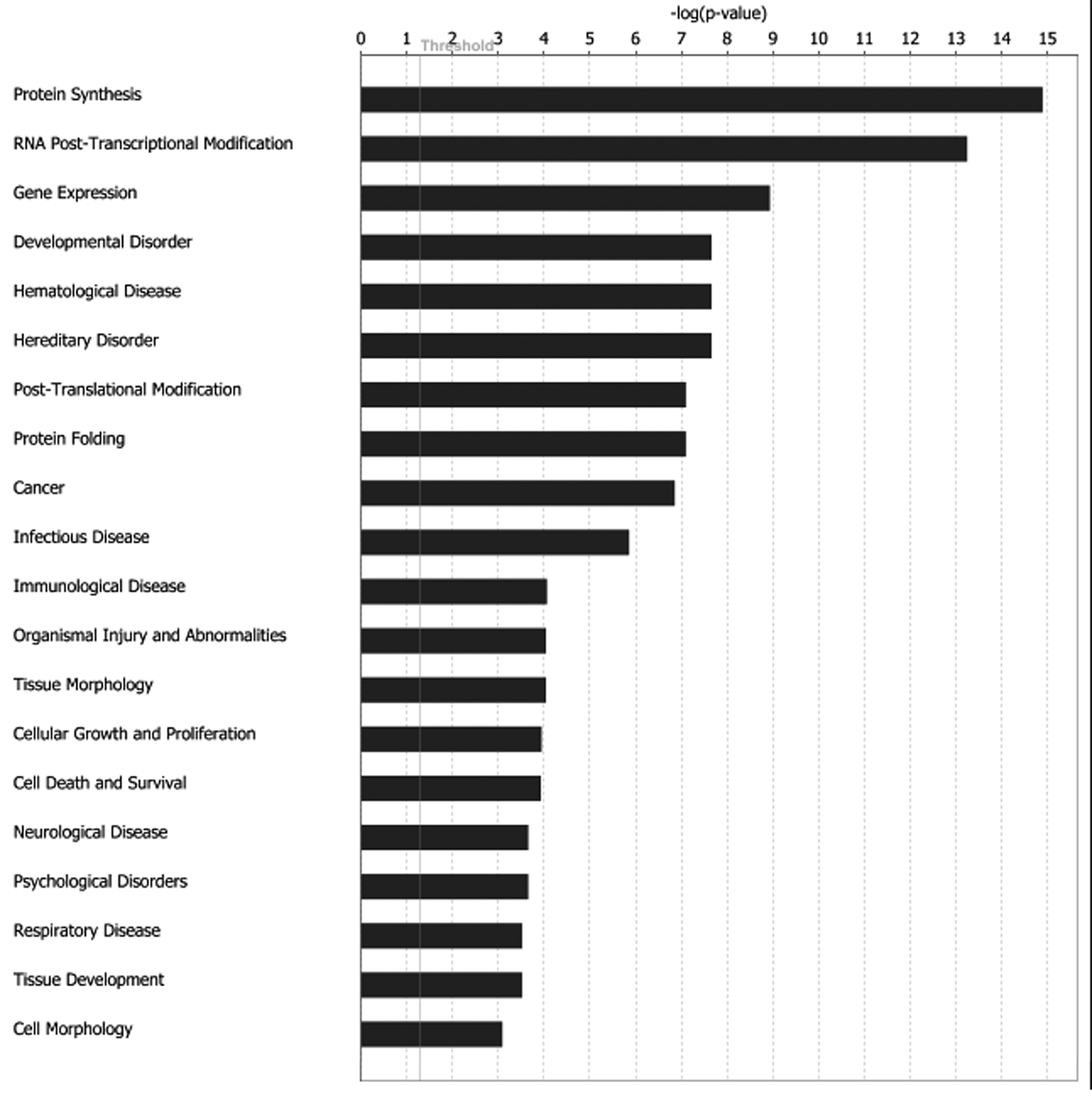

Supplement: Additional file 5: Figure s1. — Pearson correlation coefficient plot of each two proteomic runs related to the tissue specimen in each group. The three graphs in the first row of the figure refers to Pearson coefficient of any two samples in PsPD sample group (ranged from 0.974 to 0.980); The three graphs in the second row of the figure refers to the Pearson coefficient of any two samples in GBM sample group (ranged from 0.967 to 0.978). [file 12953_2015_66_MOESM5_ESM.docx]
