# Supplementary material for: A preliminary quantitative proteomic analysis of glioblastoma pseudoprogression
Source: Proteome Sci. 2015 Mar 12;13:12. doi: 10.1186/s12953-015-0066-5 (PMC4393599; doi:10.1186/s12953-015-0066-5)
Supplement: Additional file 9: Table S1. — Parameters of ROC curve for four proteins. [file 12953_2015_66_MOESM9_ESM.docx]

| Table s1 Parameters of ROC curve for four proteins | | | |
| --- | --- | --- | --- |
| Proteins | Area Under Curve | Standard Error | 95% Confidence Interval |
| ELAVL1 | 0.86* | 0.0893 | 0.614-0.975 |
| HNRNPK | 0.75 | 0.116 | 0.497-0.922 |
| FBLN1 | 0.66 | 0.137 | 0.406-0.864 |
| CDH2 | 0.9* | 0.076 | 0.662-0.988 |
| CDH2 & ELAVL1 Combined | 0.92* | 0.076 | 0.696-0.995 |

** P<0.05*

| Table s2 Pairwise comparison of ROC curves | |  |  |  |  |
| --- | --- | --- | --- | --- | --- |
| Comparisons | Difference between Areas | Standard Error | 95% Confidence Interval | Z Statistic | Significance Level |
| ELAVL1 vs. HNRNPK | 0.104 | 0.137 | -0.164-0.372 | 0.759 | 0.448 |
| ELAVL1 vs. FBLN1 | 0.195 | 0.146 | -0.092-0.481 | 1.333 | 0.182 |
| ELAVL1 vs. CDH2 | 0.039 | 0.102 | -0.161-0.239 | 0.382 | 0.703 |
| HNRNPK vs. FBLN1 | 0.091 | 0.143 | -0.190-0.372 | 0.634 | 0.526 |
| HNRNPK vs. CDH2 | 0.143 | 0.125 | -0.102-0.388 | 1.143 | 0.253 |
| FBLN1 vs. CDH2 | 0.234 | 0.136 | -0.033-0.501 | 1.715 | 0.086 |

| Table s3 IPA analysis of the significantly fold changed proteins associated with major networks and processes | | | | |
| --- | --- | --- | --- | --- |
| Network ID | Top Diseases and Functions | Associated Molecules | Score | Focus Molecules |
| 1 | Cellular Assembly and Organization, DNA Replication, Recombination, and Repair, RNA Post-Transcriptional Modification | Actin**,AIMP1,ARHGDIA,CC2D1A,CPSF6,DARS,FBL,FTH1,G3BP2,HNRNPC,HNRNPM,HNRNPU,IL1RAP,ITGB8,KARS**,NFkB (complex),**NFYC**,p85 (pik3r),**PCBP1,PDCD5**,PP2A, **PPP4R1,PRKAA1,PRMT1,RAB31,RFTN1,RPL6,RPL12,RPS3,RUVBL1,RUVBL2,SH3GLB2,TMPO,TNPO2,XRCC5** | 41 | 31 |
| 2 | Gene Expression, Protein Synthesis, DNA Replication, Recombination, and Repair | **APEX1,CBX5**,CD3,**DAB2IP**,E2f,**EEF2,EEF1A**1,Histone h3,Histone h4,**IL4I1**,Jnk,**MRPS9**,**NASP,PCNA,PFDN6,**  **PHB2,POLDIP2**,Rnr,**RPL10A,RPS16,RPS21,RPS26,RPS15A,RPS3A,SF3A1,SF3B1**,**SIRT3,SMARCA5,SMARCC2,SRRM2**,STAT5a/b,TCR,**THRAP3,TRIM28,YBX3** | 33 | 27 |
| 3 | Protein Synthesis, Lipid Metabolism, Molecular Transport | **CA3, DIABLO,EIF2A,EIF5A**,Erm,Focal adhesion kinase,  **HCFC1,HM13,HNRNPK**,Hsp27,Hsp70,Hsp90,**IGSF8,KHSRP,LARP1,LASP1,LIMA1,LPP,MSH2**,MTORC1,P38 MAPK,p70 S6k,PI3K (complex),**PPP3CB,PRUNE,PTGFRN,RBP1,**  **RP2**,**RPA2, RPL32,RPS6,RPS20,TIAL1,TUFM,VCAN** | 31 | 26 |
| 4 | Cellular Movement, Cellular Growth and Proliferation, Cell-To-Cell Signaling and Interaction | **ADAM9**,Alpha catenin,**ANP32A,BSG,C4A/C4B**,calpain,  **CDH1,CDH2,ELAVL1**,ERK1/2,**EZR,F11R,FBLN1,GLIPR2,GNA12,ITGAV**,JINK1/2,LDL,**LRP1**,Mapk,**NCL**,PI3K (family),  **PLD2**,**PLXNB2,PNN,PODXL,PPIB,PPP1R12A,PTPRZ1**,Ras,**RHOA**,Rock,**SCRIB,SET**,Tgf beta | 29 | 25 |
| 5 | Cancer, Cell Death and Survival, Tumor Morphology | 26s Proteasome,**ABCE1**,Akt,**ALG2**,Alpha tubulin,**APH1A,**  **ASCC2,BAX,CASP3**,caspase,Cg,**CLIP1,DDR1,DDX5,EIF2AK2,EIF4B,EIF4G1,ENAH**,ERK,**FNTB,FUBP1,GPAA1**,Interferon alpha,mediator,Mek,**NMT1,PIK3CB,PRKDC,**  **PUF60,RPLP2,RPS25,TARDBP,TPD52**,Vegf,**ZYX** | 29 | 25 |

*Significantly fold changed proteins from "File s5" were used for the analysis and are shown in bold type. The top 5 networks are shown. The first column indicates the network ID of the 5 networks; the second column shows the name of the top disease and functions; the third column refers to the associated molecules of the specific network; the fourth column refers to the score of each specific network; the fifth column refers to the number of the focus molecules in each network.*

| Table s4 Details on patients' age, sex and tumor location | | | | | |
| --- | --- | --- | --- | --- | --- |
|  | Histopothology | Sex | Age(years) | Location | Validation |
| MS Analysis | |  |  |  |  |
|  | GBM | male | 47 | RF | IHC & WB |
|  | GBM | female | 55 | RP | IHC & WB |
|  | GBM | female | 44 | LF | IHC & WB |
|  | PsPD | male | 45 | RP | IHC & WB |
|  | PsPD | female | 50 | RF | IHC & WB |
|  | PsPD | female | 42 | LF | IHC & WB |
| Validation |  |  |  |  |  |
|  | GBM | female | 26 | RF | IHC & WB |
|  | GBM | male | 56 | RF | IHC & WB |
|  | GBM | female | 44 | LF | IHC & WB |
|  | GBM | female | 59 | RF | IHC & WB |
|  | GBM | male | 56 | RPO | IHC & WB |
|  | GBM | female | 42 | RF | IHC & WB |
|  | GBM | female | 44 | LF | IHC & WB |
|  | GBM | male | 39 | LF | IHC & WB |
|  | PsPD | female | 61 | LFT | IHC & WB |
|  | PsPD | male | 43 | LF | IHC & WB |
|  | PsPD | male | 40 | LFP | IHC & WB |
|  | PsPD | male | 52 | RT | IHC & WB |
|  | PsPD | female | 46 | LF | WB |
|  | PsPD | male | 28 | LF | WB |
|  | PsPD | female | 51 | RF | WB |
|  | PsPD | male | 32 | LF | WB |
| *This table shows the clinical details of each sample, including the histopathological results, sex, age, tumor location and validation methods. RF, right frontal lobe; RP, right parietal lobe; RT, right temporal lobe; LF, left frontal lobe; IHC, immunohistochemical staining; WB, western blotting* | | | | | |
